# Temporal variation of the temperature-mortality association in Spain: a nationwide analysis

**DOI:** 10.1186/s12940-022-00957-6

**Published:** 2023-01-13

**Authors:** Dariya Ordanovich, Aurelio Tobías, Diego Ramiro

**Affiliations:** 1 Institute of Economy, Geography y Demography (IEGD), Spanish National Research Council (CSIC), Madrid, Spain; 2 Institute of Environmental Assessment and Water Research (IDAEA), Spanish National Research Council (CSIC), Barcelona, Spain

**Keywords:** Adaptation, Temperature, Climate change, Time-series regression, Distributed lag non-linear models

## Abstract

**Background:**

Although adaptation to continuously rising ambient temperatures is an emerging topic and has been widely studied at a global scale, detailed analysis of the joint indicators for long-term adaptation in Spain are scarce. This study aims to explore temporal variations of the minimum mortality temperature and mortality burden from heat and cold between 1979 and 2018.

**Methods:**

We collected individual all-cause mortality and climate reanalysis data for 4 decades at a daily time step. To estimate the temperature-mortality association for each decade, we fitted a quasi-Poisson time-series regression model using a distributed lag non-linear model with 21 days of lag, controlling for trends and day of the week. We also calculated attributable mortality fractions by age and sex for heat and cold, defined as temperatures above and below the optimum temperature, which corresponds to the minimum mortality in each period.

**Results:**

We analysed over 14 million deaths registered in Spain between 1979 and 2018. The optimum temperature estimated at a nationwide scale declined from 21 °C in 1979–1988 to 16 °C in 1999–2008, and raised to 18 °C in 2009–2018. The mortality burden from moderate cold showed a 3-fold reduction down to 2.4% in 2009–2018. Since 1988–1999, the mortality risk attributable to moderate (extreme) heat reduced from 0.9% (0.8%) to 0.6% (0.5%). The mortality risk due to heat in women was almost 2 times larger than in men, and did not decrease over time.

**Conclusion:**

Despite the progressively warmer temperatures in Spain, we observed a persistent flattening of the exposure-response curves, which marked an expansion of the uncertainty range of the optimal temperatures. Adaptation has been produced to some extent in a non-uniform manner with a substantial decrease in cold-related mortality, while for heat it became more apparent in the most recent decade only.

**Supplementary Information:**

The online version contains supplementary material available at 10.1186/s12940-022-00957-6.

## Introduction

The health effects of exposure to non-optimal ambient temperatures have been extensively studied [1–3]. The interest in this topic has been expressed not only by scientific community but also by health care professionals and policymakers. The alarming rates of the global warming raise a substantial concern for adverse health outcomes to aggravate in response to the amplified exposure to continuously changing and increasingly extreme temperatures [4]. The southern Mediterranean region is becoming a major hotspot due to the persistent warming of the air temperatures [5]. In particular, Spain is now one of the countries most impacted by increasing temperatures and heat waves. The progressive increase in the average annual and seasonal values of air temperatures in Spain is presented in all the projections used for the period 2081–2100 [4]. For maximum temperatures, the rise in the annual scale is predicted to be between 2.0 and 3.4 °C under the RCP4.5 scenario, while for the minimum temperatures the expected increment ranges from 1.7 to 2.9 °C under the same intermediate pathway [6].

Adaptation can happen naturally through physiological or behavioral adjustment. It can also be planned through public health initiatives like heat health warning systems, or socioeconomic development, such as improvement of living conditions [7]. Several methods have been suggested to quantify adaptation. The temperature-mortality association has been described as a J- or U-shaped curve, with the minimum being the temperature at which the mortality risk is the lowest [1, 2]. Therefore, absolute or relative shifts of the minimum mortality temperature (MMT) as a threshold from the epidemiological exposure-response function is an important indicator of how quickly populations can adapt to climate change in the long term [7, 8]. Similar to this, adaptation has also been measured by absolute or relative reductions in the risk of mortality due to non-optimal temperatures [7, 9]. However, despite the fact that adaptation might raise the MMT to compensate for part of the mortality brought on by rising temperatures, an examination of adaptation patterns should take into account whether the MMT as well as the temperature effect have changed over time, or if only one has [7, 10].

Several studies have reported that MMTs could continue to rise with increasing temperatures locally [11] and nationwide [8, 12, 13], suggesting partial adaptation to increasing temperatures. On the other hand, there has been a documented decline in the risk of heat-related mortality despite the observed rise in temperatures and the increasing frequency, severity, and length of extreme heat events [14–16]. Others have also demonstrated that the risk of cold-related mortality has recently decreased [17]. However, a thorough examination of the temporal evolution of joint indicators for long-term adaptation, such as MMT and the attributable risk related to hot and cold temperatures, especially among susceptible groups by age and gender, is seen in the literature on a rare occasion [18, 19] .

We aim to explore the long-term adaptation to non-optimal temperatures in Spain at the national level by estimating the temporal variations of the MMT and the mortality burden from heat and cold across a 40-year period, from 1979 to 2018.

## Methods

### Mortality data

We collected nationwide daily counts of all-cause mortality. Data were provided as microdata files, including sex and age, from Vital Statistics by the Spain National Institute of Statistics (INE) for the study period between January 1, 1979 and December 31, 2018.

### Temperature data

As the main source for ambient air temperature exposure, we gathered the European Centre for Medium-Range Weather Forecasts (ECMWF) reanalysis data, which comprises a combination of observations with past short-range weather forecasts rerun with weather prediction models. In particular, global atmospheric reanalysis ERA-Interim data set was retrieved from 1979 onward at a 0.125°×0.125°resolution [20, 21]. ERA-Interim uses a fixed version of a numerical weather prediction system to produce highly accurate reanalyzed data [22]. The results of the multivariate atmospheric reanalysis contained in the ERA-Interim data set have passed through quality control and multiple bias correction compared to the preceding reanalysis data sets as noted by [20]. Moreover, it has been shown that ERA-5 reanalysis data allow estimating the health effects of temperature in Spain, even in areas far or free from weather stations. Royé et al. [23] found a similar shape of the overall cumulative exposure-response curves using weather station temperature and ERA-5 reanalysis data across the full range of temperatures.

To calculate the daily (24 h) average temperature, we passed the data from the original spatial grid to the core study grid of 10 km×10 km, created according to the Infrastructure for Spatial Information in Europe (INSPIRE) technical guidelines on geographical grids. The centroid of the core grid was assigned the hourly values from the closest spatial neighbor from the ERA-Interim grid with an average distance between points estimated at 4.7 km.

### Statistical analysis

To evaluate changes in the MMT and the mortality burden from heat and cold, we split the study period into equal 10-year intervals (i.e., 1979–1988, 1989–1998, 1999–2008, and 2009–2018). For each period, we fitted a quasi-Poisson time-series regression model [24] using a distributed lag non-linear model to estimate the temperature-mortality association [25]. In particular, we controlled for seasonal and long-term trends using a natural cubic spline of time with 10 degrees of freedom per year and indicator variables for the day of the week. We used a natural cubic spline with three internal knots placed at the 10th, 75th, and 90th percentiles of the temperature distribution and the lag-response, up to 21 days, with a natural cubic spline with 3 internal knots placed at equally spaced values in the log scale (Additional file 1). The overall effects further reported in this study are computed by summing the lag-specific contributions [25]. The entire analysis was performed in R 4.2.0, and for the statistical modelling was used the *dlnm-package* [26].

These modelling choices are based on extensive previous work using an overlapping data set and have been thoroughly tested by sensitivity analyses [27, 28]. We identified the MMT from each estimated curve representing the overall cumulative exposure-response (the net effect across lags), together with an approximate parametric bootstrap estimator of its confidence interval and standard error [27]. We also calculated the MMT percentile (MMTP), defined as the percentile of the temperature distribution corresponding to the MMT. Finally, we estimated the attributable fractions of mortality [29] due to moderate cold and heat, defined at the 5th and 95th percentiles (*P*_5_ and *P*_95_) of the temperature distribution, compared to the MMT of each period. We also estimated the attributable fractions due to extreme temperatures by comparing the 1st and 99th percentiles (*P*_1_ and *P*_99_) versus the *P*_5_ and *P*_95_, respectively.

## Results

We analysed 14 203 959 deaths registered in Spain between 1979 and 2018. Table 1 shows a substantial increase in the temperature by decade: mean temperatures increased by 1.2 °C while the variability intensified by 0.3 °C. The extreme temperatures for cold (*P*_1_) and heat (*P*_99_) also increased by 0.6 and 1.4 °C, respectively (Additional file 2).

**Table 1:** Descriptive statistics for mortality counts and temperature (°C) in Spain between 1979 and 2018

			Cold		Heat	
	Total deaths	Temperature mean (sd)	P_1_	P_5_	P_95_	P_99_
1979–1988	3 014 169	14.1 (5.8)	3.7	6.2	23.8	25.0
1989–1998	3 412 755	14.4 (5.7)	4.8	6.7	24.6	25.9
1999–2008	3 747 522	15.0 (6.0)	4.3	6.3	24.6	26.0
2009–2018	4 029 513	15.3 (6.1)	4.3	6.6	25.1	26.4

**Fig. 1:**
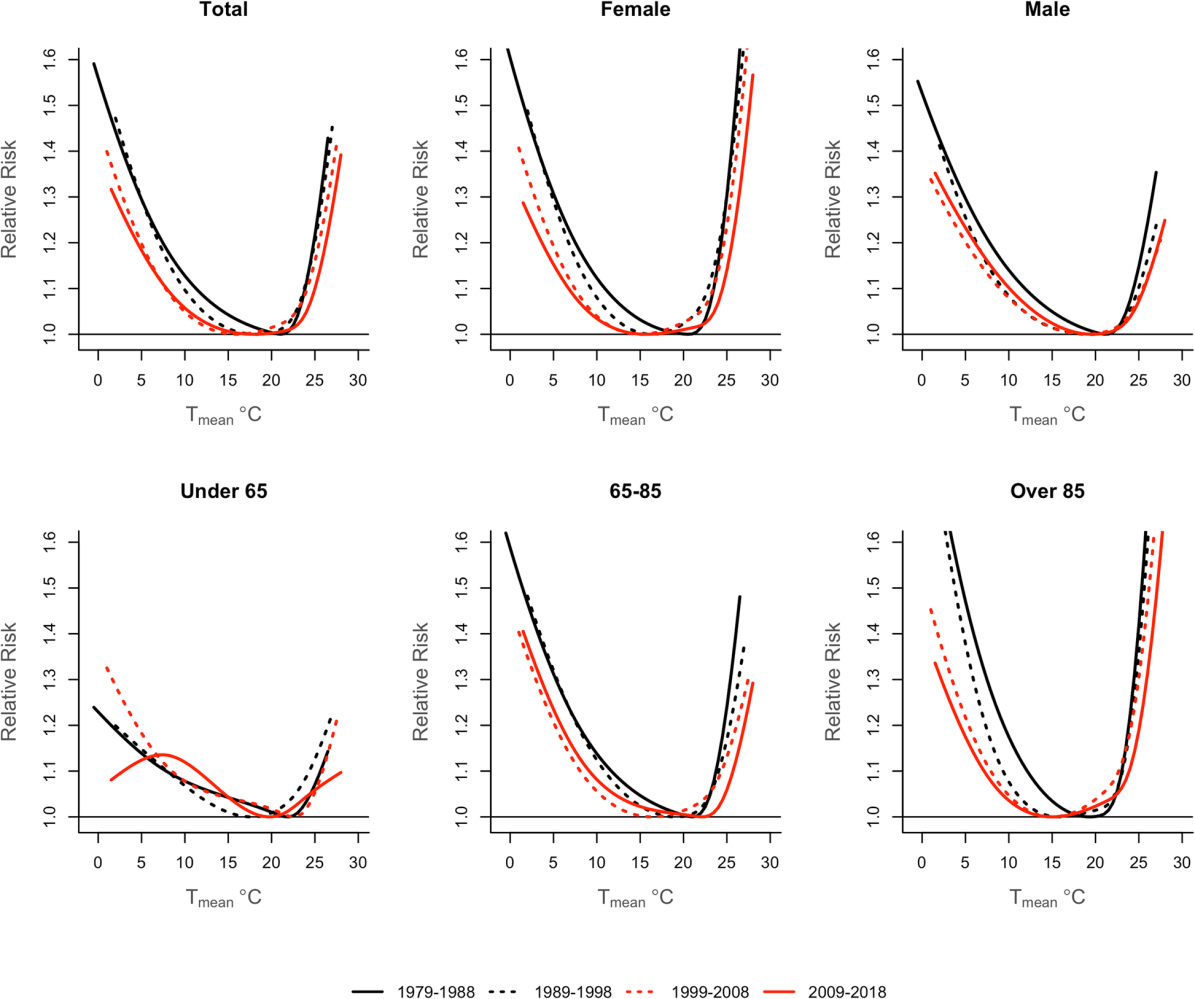
Overall temperature-mortality associations in Spain by decade between 1979 and 2018

**Fig. 2:**
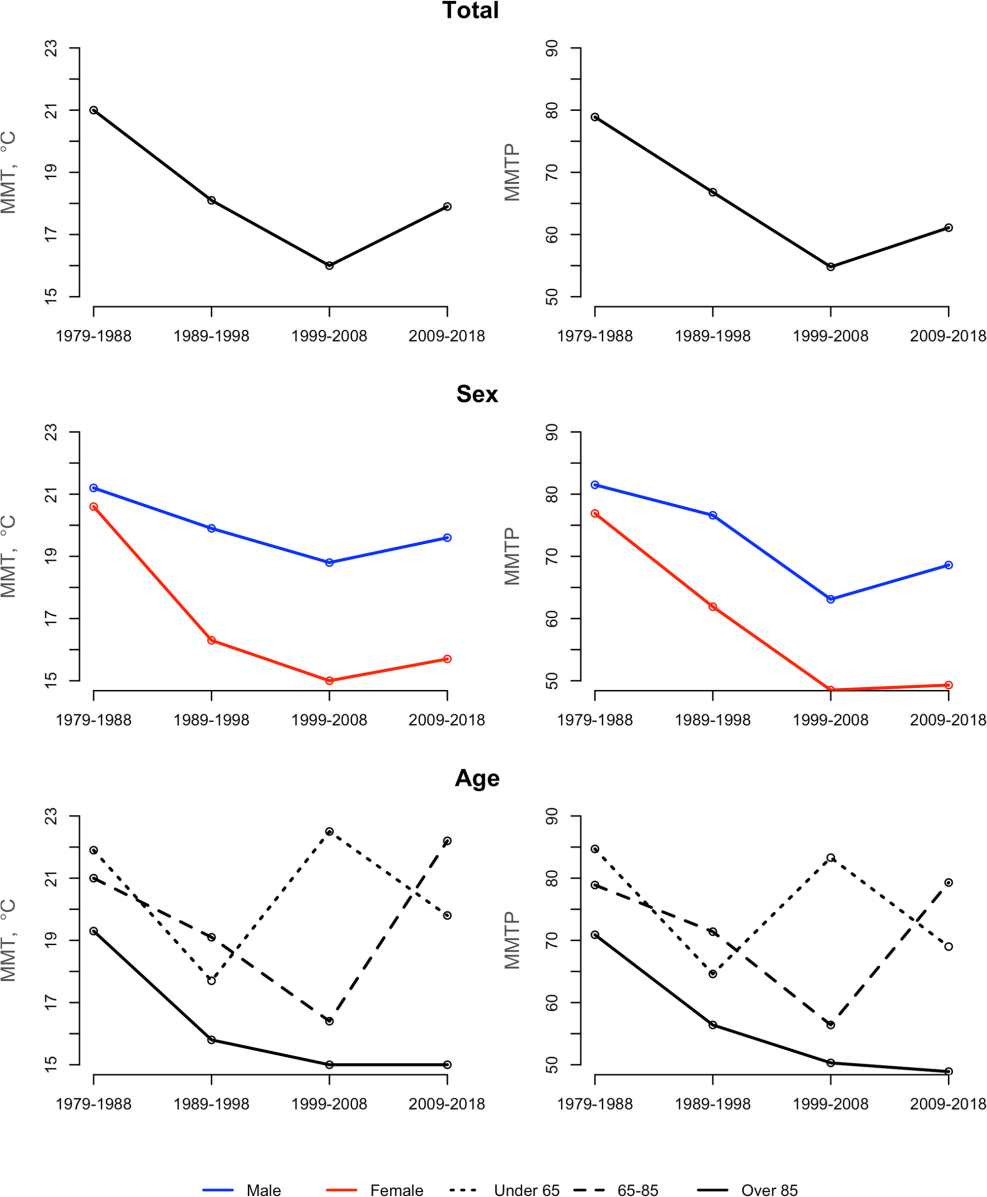
Temporal evolution in the MMT and MMTP in Spain by decade between 1979 and 2018

The temperature-mortality association shifted from a V-shape in 1979–1988 to a U-shape in 2009–2018, revealing a progressive flattening of the exposure-response curve (Fig. 1). The MMT exhibited a non-uniform pattern (Fig. 2). The maximum MMT was registered in the 1979–1988 decade (21.0 °C), declined in 1989–1999 and 1999–2008 (18.1 °C and 16.0 °C, respectively), and raised in 2009–2018 (17.9 °C) (Table 2).

**Table 2:** MMT,°C and MMTP,% in Spain by decade between 1979 and 2018

	1979–1988	1989–1998	1999–2008	2009–2018
MMT				
Total	21.0 (19.3–21.5)	18.1 (16.8–20.5)	16.0 (14.4–18.0)	17.9 (15.5–22.0)
Sex				
Male	21.4 (19.4–21.9)	20.5 (17.6–21.7)	18.0 (15.6–22.1)	19.7 (17.4–22.4)
Female	20.7 (17.8–21.4)	17.1 (15.8–18.5)	14.5 (13.0-16.4)	15.1 (12.8–21.9)
Age				
Under 65	21.9 (16.5–22.7)	17.7 (15.0-20.7)	22.5 (14.0-23.3)	19.8 (18.0-22.4)
65–85	21.0 (19.0-21.6)	19.1 (17.6–20.9)	16.4 (14.5–19.4)	22.2 (16.2–22.9)
Over 85	19.3 (17.1–21.2)	15.8 (14.0–21.0)	15.0 (13.2–16.9)	15.0 (12.9–17.8)
MMTP				
Total	78.9 (70.9–82.0)	66.8 (60.7–76.6)	54.8 (48.1–63.1)	61.1 (50.7–78.3)
Sex				
Male	81.5 (71.2–84.7)	76.6 (64.3–81.3)	63.1 (52.8–81.1)	68.6 (58.9–80.3)
Female	76.9 (65.3–81.5)	61.9 (56.4–68.5)	48.5 (42.0-56.4)	49.3 (39.1–77.8)
Age				
Under 65	84.7 (60.9–89.3)	64.6 (52.9–77.5)	83.3 (46.6–88.1)	69.0 (61.6–80.3)
65–85	78.9 (69.4–82.6)	71.4 (64.3–78.2)	56.4 (48.5–68.2)	79.3 (53.7–83.3)
Over 85	70.9 (63.0-79.9)	56.4 (47.7–78.6)	50.3 (43.2–58.5)	48.9 (39.6–60.6)

In terms of the demographic strata, the change in the temperature-mortality shape was also observed by sex and age (Fig. 2). However, women had remarkably lower MMT than men, with a maintained gap of approximately 3.5 °C from 1989 to 1999 and a declining trend until the last decade (Table 2). As for the age group, the eldest individuals (85+) had the lowest MMTs, and a more pronounced declining trend than those aged 65–85, who showed a considerable MMT increase of 5 °C in 2009–2018.

The mortality burden from moderate cold shows an almost 3-fold reduction, from 6.1% in 1979–1988 to 2.4% in 2009–2018. Similarly, mortality burden from extreme cold reduced from 1.1 to 0.7% (Table 3). The mortality risk attributable to heat shows a smaller reduction than cold, but from 1988 to 1999 onward. Moderate heat is reduced from 0.9 to 0.6%, while extreme heat from 0.8 to 0.5%. The attributable fraction associated with moderate cold decreased more substantially in women (from 5.5% in 1979–1988 to 1.4% in 2009–2018) than in men (6.6–3.8%). The impact of extreme cold was similar for both sexes until 2009–2018 when it decreased more in women (0.6%) than in men (0.8%). Oppositely, the mortality risk attributable to moderate and extreme heat was almost 2 times larger in women and did not decrease over time (Table 3). By age group, the impact of moderate cold was reduced mainly in the elderly (7.9–1.6%) and in those aged 65–85 (6.5–3.6%). A similar pattern was found for extreme cold. Attributable fractions associated with moderate and extreme heat exposure were higher in the eldest individuals than in other age groups throughout the entire study period.

**Table 3:** Mortality attributable fractions (%) due to heat and cold in Spain between 1979 and 2018

	Total	Sex		Age		
		Female	Male	Under 65	65–85	Over 85
*1979–1988*						
* P*_1_ *vs. P*_5_	1.12 (0.93–1.31)	1.12 (0.90–1.36)	1.11 (0.86–1.36)	0.52 (0.19–0.82)	1.20 (0.99–1.43)	1.78 (1.43–2.11)
* P*_5_ *vs. MMT*	6.06 (3.92–7.96)	5.51 (3.10–7.82)	6.62 (4.03–9.04)	4.03 (0.07–7.37)	6.54 (4.10–8.87)	7.85 (4.81–10.54)
* MMT vs. P*_95_	0.34 (0.23–0.46)	0.52 (0.32–0.70)	0.20 (0.11–0.31)	0.08 (-0.01-0.17)	0.37 (0.23–0.50)	0.79 (0.22–1.33)
* P*_95_ *vs. P*_99_	0.49 (0.41–0.56)	0.67 (0.57–0.78)	0.32 (0.23–0.42)	0.17 (0.04–0.29)	0.52 (0.43–0.62)	0.88 (0.69–1.06)
*1989–1998*						
* P*_1_ *vs. P*_5_	0.97 (0.86–1.09)	1.07 (0.93–1.22)	0.90 (0.73–1.05)	0.51 (0.30–0.72)	1.07 (0.92–1.21)	1.20 (1.02–1.38)
* P*_5_ *vs. MMT*	3.87 (2.84–4.87)	3.98 (2.73–5.15)	4.12 (2.20–5.92)	2.59 (0.70–4.41)	4.98 (3.51–6.37)	3.24 (1.94–4.59)
* MMT vs. P*_95_	0.89 (0.52–1.26)	1.56 (0.97–2.15)	0.40 (0.17–0.63)	0.92 (0.07–1.77)	0.71 (0.33–1.08)	1.42 (0.52–2.26)
* P*_95_ *vs. P*_99_	0.75 (0.62–0.87)	1.10 (0.91–1.26)	0.45 (0.32–0.58)	0.50 (0.24–0.76)	0.63 (0.48–0.78)	1.20 (0.98–1.42)
*1999–2008*						
* P*_1_ *vs. P*_5_	0.76 (0.65–0.88)	0.76 (0.62–0.90)	0.79 (0.63–0.95)	0.64 (0.32–1.01)	0.80 (0.64–0.95)	0.88 (0.71–1.06)
* P*_5_ *vs. MMT*	2.21 (1.41–2.94)	1.78 (0.94–2.54)	2.92 (1.64–4.22)	3.97 (-0.64-8.10)	2.47 (1.36–3.52)	2.24 (1.16–3.29)
* MMT vs. P*_95_	1.10 (0.39–1.77)	1.94 (0.97–3.02)	0.53 (-0.12-1.16)	0.12 (-0.01-0.25)	1.03 (0.15–1.82)	2.22 (1.05–3.31)
* P*_95_ *vs. P*_99_	0.59 (0.47–0.71)	0.89 (0.71–1.06)	0.34 (0.20–0.49)	0.24 (0.10–0.37)	0.48 (0.33–0.63)	1.01 (0.82–1.21)
*2009–2018*						
* P*_1_ *vs. P*_5_	0.68 (0.54–0.82)	0.57 (0.44–0.71)	0.81 (0.62–1.01)	0.50 (0.16–0.79)	0.83 (0.60–1.05)	0.66 (0.50–0.82)
* P*_5_ *vs. MMT*	2.38 (1.23–3.48)	1.37 (0.54–2.27)	3.76 (1.99–5.49)	4.56 (1.65–7.28)	3.57 (1.37–6.02)	1.62 (0.64–2.54)
* MMT vs. P*_95_	0.64 (0.19–1.09)	1.12 (0.14–2.01)	0.43 (0.00-0.84)	0.62 (-0.12-1.44)	0.25 (0.11–0.39)	1.63 (0.67–2.58)
* P*_95_ *vs. P*_99_	0.49 (0.39–0.59)	0.69 (0.53–0.84)	0.33 (0.21–0.44)	0.25 (0.05–0.44)	0.33 (0.25–0.41)	0.82 (0.65–0.99)

Cold: moderate cold (*P*_5_ *vs. MMT*) and extreme cold (*P*_1_ *vs. P*_5_)

Heat: moderate heat (*MMT vs. P*_95_) and extreme heat (*P*_95_ *vs. P*_99_)

## Discussion

This study examined the temporal variation in the ambient temperature-mortality association across a 40-year period in Spain. We found a flattening of the exposure-response curve with a considerable decrease in the cold-related mortality compared to the heat. Women experienced the most marked reduction in the cold-related mortality, being generally less affected than men. Oppositely, men were less impacted by the heat. The elderly also experienced the largest reduction in the both cold- and heat-related mortality. The MMT decreased until the most recent decade, 2009–2018, when it increased by almost 2 °C. We observed a similar pattern by sex, although women had much lower MMTs. The elderly also had generally lower MMTs with a pronounced declining trend over time, while those aged 65–85 showed a considerable increase of 5 °C in the last decade.

Overall, our results agree with previous studies showing that most of the mortality burden was caused by days colder than the MMT compared to warmer days. The contribution of extreme days was comparatively lower than moderately hot and cold temperatures [2]. However, others have also examined temporal changes in the temperature-mortality association. We also found similar results to those previously reported in Spain. Vicedo-Cabrera et al. [30], as part of a multi-country assessment on potential adaptive mechanisms to cold and heat, reported a decrease in the cold-related mortality for Spain and a less noticeable decrease in the heat-related mortality. Martínez-Solanas et al. [31] compared the temporal changes between two decades before and after the activation of the Spanish Heat Health Prevention Plan [32], reporting greater reductions in the cold- and heat-related mortality in the elderly. Achebak et al. [18] focused on the cause-specific mortality and showed that the attributable fraction of cardiovascular deaths due to the warm temperatures was higher for women, while for cold temperatures was higher in men. Vicedo-Cabrera et al. [30] reported a consistent decrease in heat-related mortality over the past decades in most of the ten countries evaluated, but the reduction in the cold mortality was only found in half of them.

Despite reporting similar results, the previous studies used a two-stage design combining city-specific estimates to derive a national assessment [33]. These studies mainly included data from the capitals of the provinces. Despite the geographic heterogeneity, we primarily focused on the national level, therefore our analysis included all daily deaths nationwide, regardless of the size of the city (i.e. including the areas with less than 10 000 inhabitants). We also accurately calculated a national average daily temperature using the ERA-interim data set, which provided a similar exposure to the Spain National Meteorology Agency calculated using 42 reference stations [34]. A recent study on the long-term adaptation to heat stress in the Netherlands also used the same nationwide approach as the one implemented in our study [12]. Nevertheless, we conducted a sensitivity analysis comparing the exposure-response curves for the temperature mortality association from our nationwide analysis and the city-specific estimates from the 52 provincial capital cities (Additional file 3) using a two-stage approach (Additional file 4).

The MMT has also been used as a threshold for climate adaptation. If populations become less susceptible to heat, an increase in the MMT can be expected over time [11, 13, 35], similar to higher MMT values in warmer cities due to geographic differences [27]. If the MMTP is fixed at a certain percentile of temperature distribution and all other factors are held constant, warmer climates would tend to increase the MMT. Similarly, if the MMT is fixed, higher temperatures would shift the MMTP to a lower percentile of the temperature distribution. Several studies have reported that MMT could continue rising with increasing temperatures at the local level and nationwide, suggesting partial adaptation to warmer temperatures. The observed increase of the MMT by 1.9 °C in the last decade (0.1 °C/year) seen in our study is similar to the estimates previously reported for Japan (0.12 °C/year) [17] and the Netherlands (0.15 °C/year), although these countries showed a sustained increase in the MMT in 1972–2012 and 1995–2017, respectively. In France the MMT increased by 0.027 °C/year for adults over 65 years in 1968–2009 [13] while in our study this parameter was estimated at 0.037 °C/year for the population aged 65–85 in 1979–2018. Although the MMT decreased since 1979 and increased in the last decade, the shape of the temperature-morality association changed substantially from a V-shape in the first decade to a U-shape in the last decade. The continuous flattening of the exposure-response curve across the decades implies a more extensive range of optimal temperatures for both cold and heat, taking into account the MMTs’ confidence intervals. Therefore, we could consider that adaptation to non-optimal temperatures in Spain has been produced progressively since 1989 for cold and since 2009 for heat. It is, however, of great importance to highlight the fact that the analysis based on all-cause mortality data might conceal some patterns otherwise discernible when working with specific causes of death. Thus, the MMTs estimated for the same period of time in Spain using cardiovascular and respiratory mortality data exhibited a multidirectional trend over time [18, 19, 36]. In case of the cardiovascular diseases, the optimum temperatures were monotonically increasing with time, which attributed to the fact that for this group of diseases the risks for both hot and cold temperatures were persistently reducing while the rate of reduction for hotter temperatures was higher. On the contrary, the MMTs for respiratory diseases were cooling down until 2000–2010, driven by a higher rate of decrease of risk of death for cold temperatures, and remained constant from then onwards. These results are in line with the findings presented in our study, which is based on the individual level mortality data scaled up to the national level. The disaggregation of these individual data by cause of death, sex and age in barely populated rural areas (municipalities with less than 10 000 inhabitants) is not available since the provider cannot release these data due to the risk of the disclosure of personal information, in this way complying with the confidentiality and data protection regulations. Moreover, the present analysis tackles the topic of the adaptation to warming environment from a wider perspective, which might affect the flexibility of the selected model to reproduce the monotonically decreasing trends in the risks at all temperatures.

Gosling et al. [7] suggested to model the adaptation by threshold shifts and reductions in the exposure-response association. In our study, we quantified the temporal variation in impact estimates such as MMTs and attributable fractions, giving a comprehensive picture of how non-optimal temperatures have affected the population over the past 40 years in Spain. Days with exceptionally high temperatures have become more common, and this trend is expected to continue. Therefore, it is reasonable to assume that, to some extent, people and societies can adapt to gradual increases in average temperatures. The decrease in the cold-related mortality could be explained by a possible biological adaptation to extreme ambient temperatures [37] or a possible modification in susceptibility to temperature [30].

The decrease in the heat-related mortality since 1999 occurred despite the progressive shift of the temperatures towards warmer ranges. It has been attributed to the implementation the Heat Health Prevention Plan [31]. Moreover, other factors may contribute to changes in susceptibility to non-optimal temperatures, such as the ageing population, improvements in healthcare and health interventions, living conditions and urban built environment, and social progress (Table 4). The proportion of population aged over 65 years increased by 6.2% between 1979 and 1989 and 2009–2018, life expectancy 6.5 years, GDP 67.5% and health expenditure was doubled. In addition, the use of air conditioning has increased in the last decades from 9 to 43.7%, which can also explain part of the decline in the heat-related mortality [33]. We acknowledge some limitations in our study. First, we considered all causes of death, including ones that might not be related to ambient temperatures, rather than just natural causes. However, this has allowed us to include all deaths in locations with less than 10,000 inhabitants that have been rarely considered in previous studies in Spain. Likewise, our study did not consider geographic variability since the main objective was to address the long-term adaptation to non-optimal temperatures at the national level. Second, we could not differentiate between physiological, behavioral, cultural, society-based and technology-driven adjustments. This is a standard limitation in epidemiological studies of long-term adaptation to climate and weather. However, as previously stated, we summarized the trends of non-climate driven factors changing over 40 years, such as demographic changes and economic growth, which could influence long-term adaptation to climate (Table 4). Third, we did not include data on air pollution and influenza epidemics. Nevertheless, Buckley et al. [38] stated that air pollution should not confound the effects of temperature. Others also showed no changes in the temperature-mortality association when fitting influenza epidemics in sensitivity analyses [39]. Finally, we accounted for short-term mortality displacement of up to three weeks. Mortality displacement at a longer scale was not considered. However, Armstrong et al. [40] reported that the effects of having low winter mortality in the following summer were low in Spain.

**Table 4:** Changes in the demographic and socioeconomic indicators in Spain between 1979 and 2018

	Population older than 65 years (%) (^a^)	Life expectancy at birth (years) (^b^)	GDP, constant 2015 US$ (^c^)	Health expenditure as % of GDP (^d^)	Air conditioning prevalence (%) (^e^)
1979–1988	11.7	76.1	15389.6	4.5	
1989–1998	14.5	77.8	19842.1	5.5	9.0
1999–2008	16.7	80.1	25463.8	7.5	27.2
2009–2018	17.9	82.6	25784.7	9.1	43.7

Indicators estimated as average value for each decade according to the data availability

^a^ Population structure indicators by National Institute of Statistics of Spain. Available at www.ine.es

^b^ Demographic indicators by National Institute of Statistics of Spain. Available at www.ine.es

^c^ GDP per capita by World Bank. Available at data.worldbank.org

^d^ Public health spending in Spain: 10 years of national health system by Ministry of Economy and Finance. Global Health Expenditure by World Health Organization. Available at apps.who.int

^e^ Population and Housing Census, Life Conditions Survey, Households and Environment Survey by National Institute of Statistics of Spain. Available at www.ine.es. Housing survey by the Center for Sociological Studies. Available at www.cis.es

## Conclusion

Our study provides nationwide quantitative estimates for long-term adaptation to non-optimal temperatures in Spain over the past 40 years. Despite the progressively warmer temperatures in Spain, we observed a persistent flattening of the exposure-response curves, which marked an expansion of the optimal temperature ranges for cold and heat. Adaptation has been produced to some extent in a non-uniform manner with a substantial decrease in cold-related mortality, while for heat became more apparent in the most recent decade only. Since the climate change projections indicate a substantial increase in temperatures in Spain [4, 6] and more than a third of all deaths caused by heat may be attributable to global warming [41], Spain must set as a priority to develop an adaptation strategy to climate change.

### Supplementary Information

**Additional file 1.** Sensitivity analysis results. Figure 6. Sensitivity analysis performed for degrees of freedom for seasonal trend (top row), for the lag period (middle row) and for the number of knots for exposure-response function (bottow row).

**Additional file 2.** Distribution of the mean temperature in Spain by decades between 1979 and 2018 in Spain. Figure 3. Variations in the mean temperature between 1979 and 2018 in Spain (including archipelagos).

**Additional file 3.** Overall temperature-mortality associations estimated for Spanish provincial capital cities between 1979 and 2018. Figure 4. Overall temperature-mortality associations estimated for Spanish provincial capital cities between 1979 and 2018.

**Additional file 4.** Comparison of the overall temperature-mortality association estimated nationwide and using a two-stage design pooling provincial capital cities exposure-response curves. Figure 5. Temperature-mortality associations estimated nationwide and using a two-stage design pooling provincial capital cities exposure-response curves.

## Data Availability

Mortality data cannot be made publicly available under the Spain National Institute of Statistics (INE) sharing agreement. Temperature data can be downloaded from the European Centre for Medium-Range Weather Forecasts (ECMWF).

## Abbreviations

RCP: Representative Concentration Pathway
MMT: Minimum Mortality Temperature
MMTP: Minimum Mortality Temperature Percentile
IPCC: Intergovernmental Panel on Climate Change
ECMWF: Centre for Medium-Range Weather Forecasts
INSPIRE: Infrastructure for Spatial Information in Europe
