# Supplementary figures and images for: Temporal variation of the temperature-mortality association in Spain: a nationwide analysis
Source: Environ Health. 2023 Jan 13;22:5. doi: 10.1186/s12940-022-00957-6 (PMC9838025; doi:10.1186/s12940-022-00957-6)

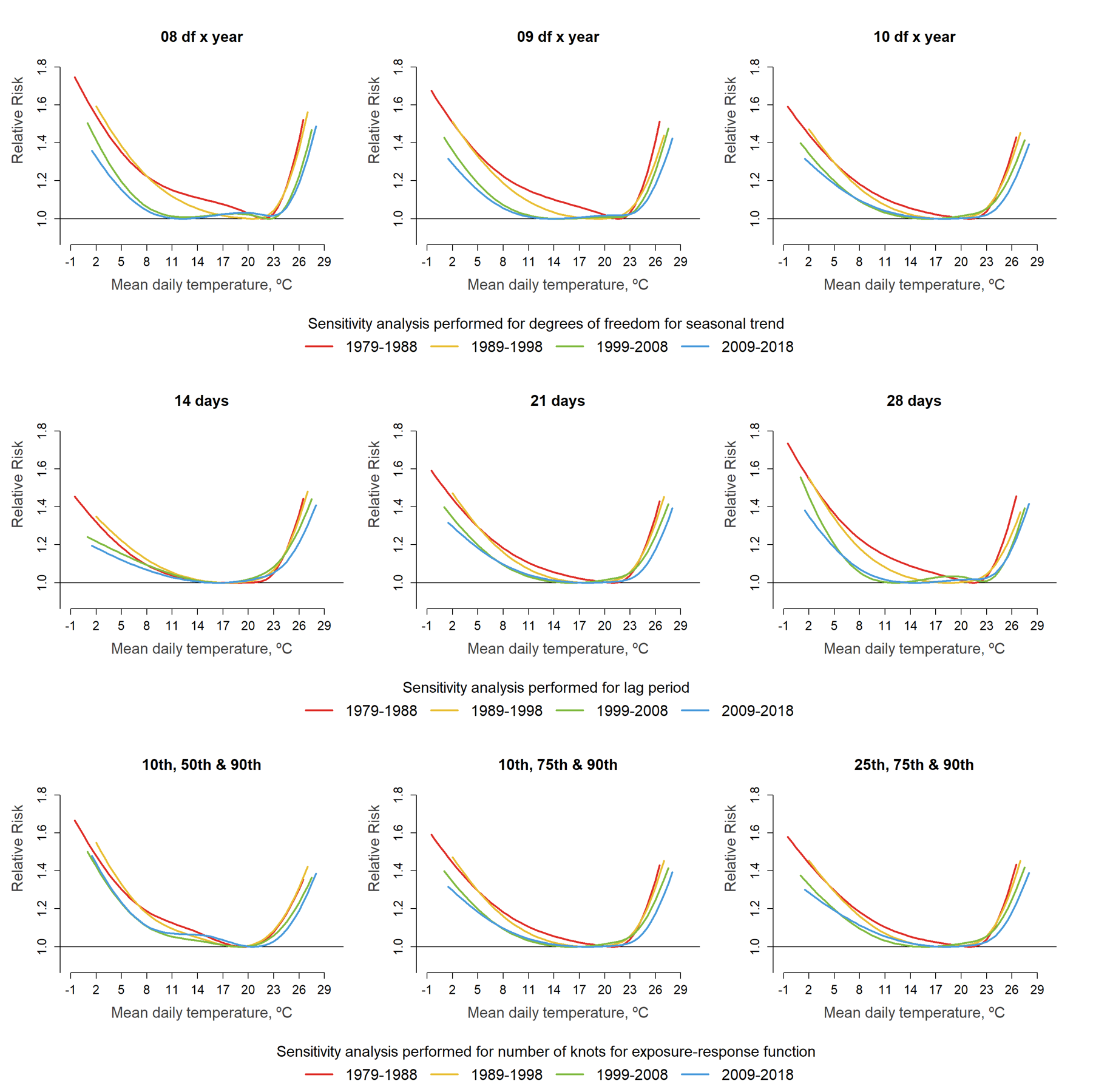

Supplement: Supplementary file 1 — Additional file 1. Sensitivity analysis results. Figure 6. Sensitivity analysis performed for degrees of freedom for seasonal trend (top row), for the lag period (middle row) and for the number of knots for exposure-response function (bottow row). [file 12940_2022_957_MOESM1_ESM.png]

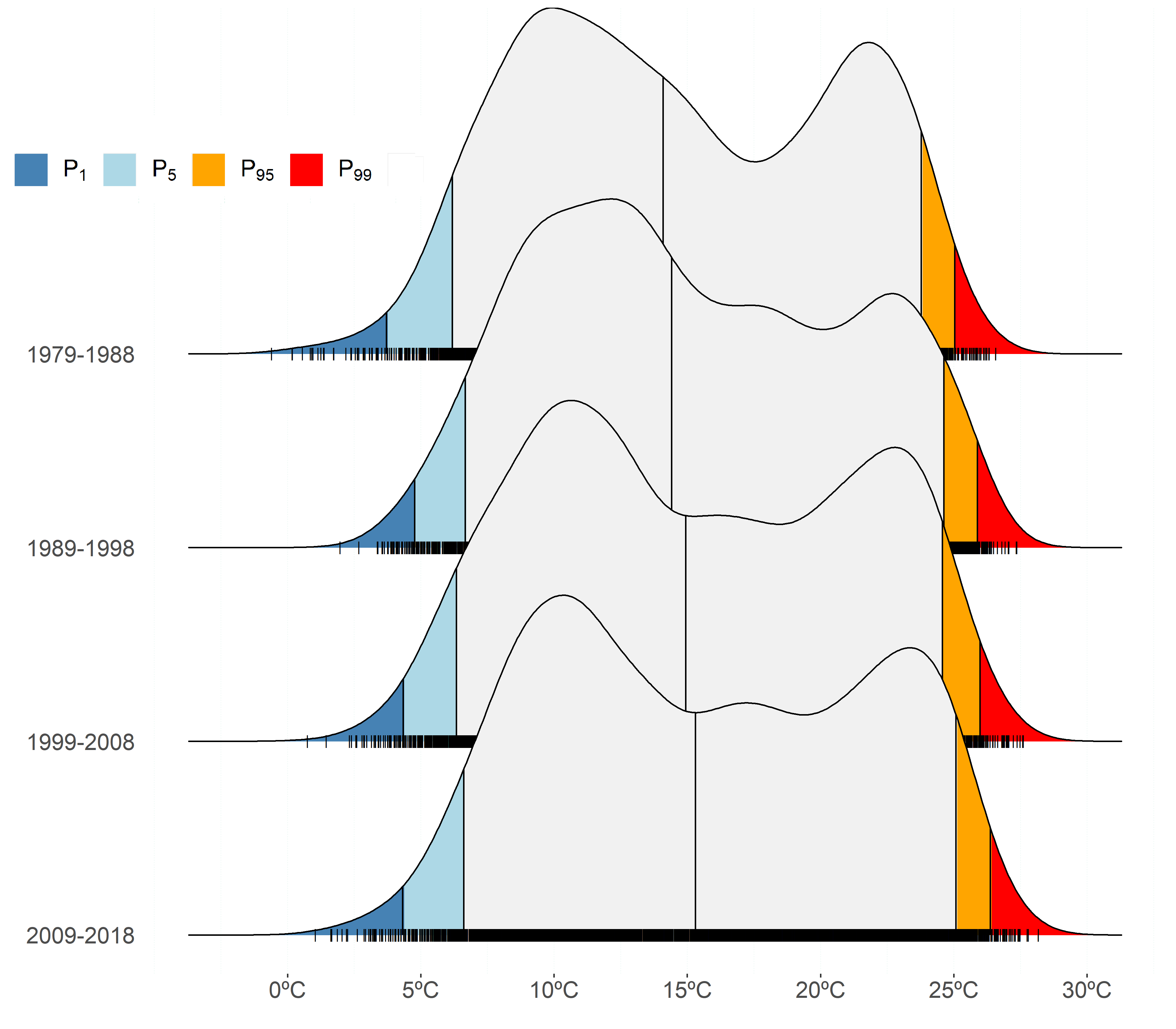

Supplement: Supplementary file 2 — Additional file 2. Distribution of the mean temperature in Spain by decades between 1979 and 2018 in Spain. Figure 3. Variations in the mean temperature between 1979 and 2018 in Spain (including archipelagos). [file 12940_2022_957_MOESM2_ESM.png]

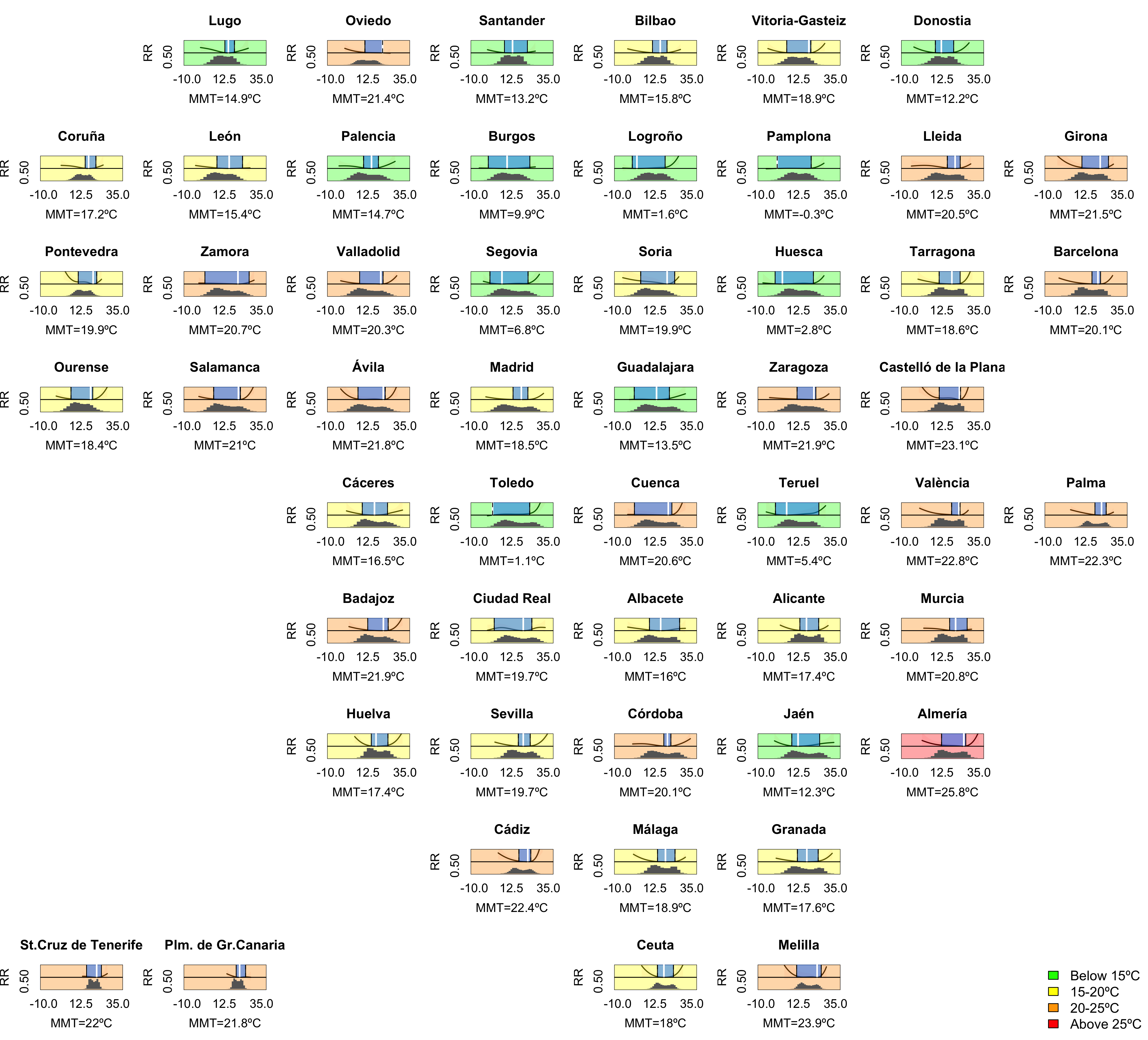

Supplement: Supplementary file 3 — Additional file 3. Overall temperature-mortality associations estimated for Spanish provincial capital cities between 1979 and 2018. Figure 4. Overall temperature-mortality associations estimated for Spanish provincial capital cities between 1979 and 2018. [file 12940_2022_957_MOESM3_ESM.png]

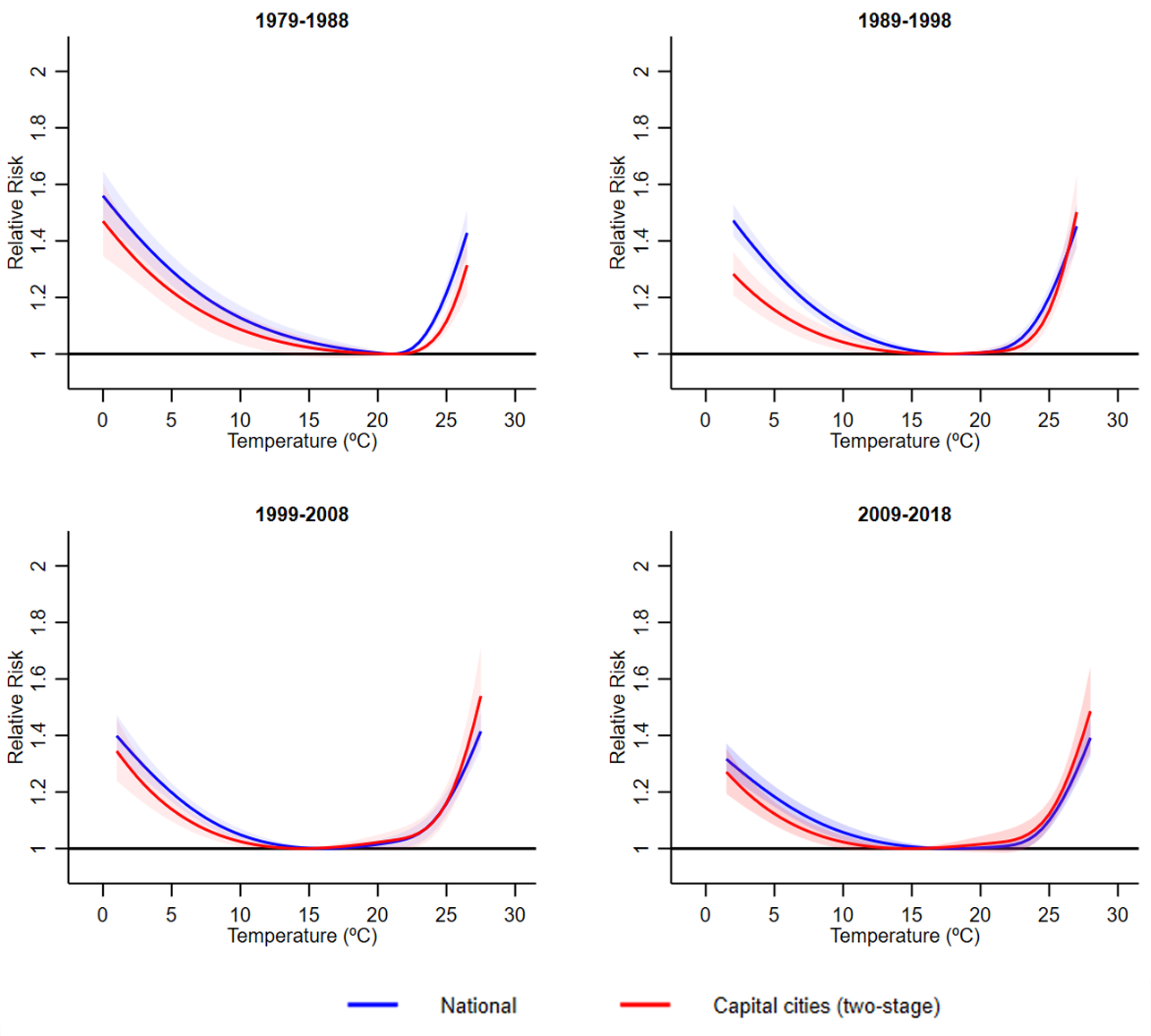

Supplement: Supplementary file 4 — Additional file 4. Comparison of the overall temperature-mortality association estimated nationwide and using a two-stage design pooling provincial capital cities exposure-response curves. Figure 5. Temperature-mortality associations estimated nationwide and using a two-stage design pooling provincial capital cities exposure-response curves. [file 12940_2022_957_MOESM4_ESM.png]
